# Chain reaction: LINC complexes and nuclear positioning

**DOI:** 10.12688/f1000research.16877.1

**Published:** 2019-01-31

**Authors:** Brian Burke

**Affiliations:** 1 Institute for Medical Biology, 8A Biomedical Grove, #06-06 Immunos , 138648, Singapore

**Keywords:** Nucleus, Nuclear positioning, Nuclear envelope, LINC complex, SUN domain, KASH domain, Nesprin, Cytoskeleton

## Abstract

Nuclear positioning plays an essential role in defining cell architecture and behaviour in both development and disease, and nuclear location frequently adjusts according to internal and external cues. For instance, during periods of migration in many cell types, the nucleus may be actively repositioned behind the microtubule-organising centre. Nuclear movement, for the most part, is dependent upon coupling of the cytoskeleton to the nuclear periphery. This is accomplished largely through SUN and KASH domain proteins, which together assemble to form LINC (linker of the nucleoskeleton and cytoskeleton) complexes spanning the nuclear envelope. SUN proteins of the inner nuclear membrane provide a connection to nuclear structures while acting as a tether for outer nuclear membrane KASH proteins. The latter contain binding sites for diverse cytoskeletal components. Recent publications highlight new aspects of LINC complex regulation revealing that the interplay between SUN and KASH partners can strongly influence how the nucleus functionally engages with different branches of the cytoskeleton.

## Introduction

The defining feature of metazoa is recruitment of diverse cell types into a broad range of tissues and organ systems. Such specialisation during development goes hand-in-hand with oftentimes extensive cellular remodelling. Think of non-contractile myoblasts fusing to form mature muscle fibres or neuroepithelial cells transforming into cortical neurons. Reorganisation of cellular architecture almost invariably involves structural and compositional changes to the cytoskeleton, repositioning of organelles, and expansion or contraction of different cellular compartments. Even the nucleus, as the largest organelle in multicellular animals, may be the object of large-scale movements ^1^. In certain cell types such as erythroblasts, it is even dispensed with entirely in a process of active ejection.

What has become increasingly clear is that the nucleus maintains extensive contacts with the cytoskeleton. In this way, it may function as a nexus for the dissemination of mechanical forces throughout the cell. It is also apparent that nuclear components may experience physical forces originating in the cytoplasm or even in the extracellular environment. This was demonstrated by Maniotis *et al*., who used ligand-coated beads to displace extracellular matrix–associated plasma membrane proteins and their coupled cytoskeletal elements ^2^. The result was distortion and dislocation of intracellular structures, including the nucleus, in the direction of bead movement. More surprising was the corresponding displacement of intranuclear landmarks such as nucleoli. Related experiments in which a microneedle was used to directly perturb cytoplasmic structures, including the cytoskeleton, gave qualitatively similar results. These conceptually simple experiments implied that there must be structures at the nuclear envelope (NE) that can mechanically couple nuclear and cytoplasmic components ^3^. The identification of such structures in the intervening years has represented an important advance in our understanding of the biology of the NE and how this contributes to the organisation of both the nucleus and the cytoplasm ^4– 7^.

## LINCing the nucleus and cytoskeleton

The interface between nuclear and cytoplasmic compartments is formed by the NE ^8^. Its most prominent features are inner and outer nuclear membranes (INM and ONM) separated by a perinuclear space (PNS). The ONM shares multiple connections with the endoplasmic reticulum to which it is functionally related. In contrast, the INM contains a unique array of integral membrane proteins that associate with nuclear components. Despite compositional differences, the INM and ONM display continuities at sites occupied by nuclear pore complexes (NPCs), massive channels that mediate movement of macromolecules back and forth across the NE. The final significant component of the NE is the nuclear lamina, a filamentous meshwork composed of A- and B-type lamins which is associated with the nuclear face of the INM and which provides structural integrity to the NE ^9, 10^.

Molecular details of force transmission between the nucleus and cytoplasm first emerged from observations that cytoplasmic dynein was recruited to the NE during G _2_ of the cell cycle ^11– 13^. This was subsequently shown to facilitate NE breakdown and dispersal during mitotic prometaphase ^14, 15^. In essence, NE-associated dynein, engaging with astral microtubules, serves to peel open the NE. However, it is obvious that, to be effective, a dynein anchor at the nuclear surface must be able to transmit forces across the nuclear membranes to the nuclear lamina. Ensuing studies implicated NPCs as just such an anchor, and two distinct NPC-associated dynein-binding sites were identified. The first of these involved the Nup107/160 complex, which is a major component of the structural framework of the NPC ^16^. The second dynein-binding site featured Nup358/RanBP2, a component of filament structures that extend from the NPC framework into the cytoplasm ^17^. In addition to NE breakdown, NPC-associated dynein is required to drive vectorial nuclear movement in certain cell types. A notable example is interkinetic nuclear migration (IKNM) in neuroepithelial cells ^16^. One aspect of IKNM is dynein-mediated basal-to-apical movement of nuclei during G _2_ of the cell cycle. This occurs as a prelude to cell division and is essential for ordered development of the central nervous system.

The role of NPCs as cytoskeletal attachment sites is restricted to the microtubule system and then only for limited periods during the cell cycle. During interphase in most somatic cells, it is LINC (linker of the nucleoskeleton and cytoskeleton) complexes that sustain this function ^1, 6^. LINC complexes are versatile structures recognised in all nucleated cells examined to date. This includes plant ^18, 19^ and animal ^20– 23^ as well as fungal ^24– 26^ cells. LINC complexes are assembled from members of two families of integral membrane proteins. SUN (Sad1, UNC-84) domain proteins are residents of the INM in which their N-terminal regions are exposed to the nuclear contents while C-terminal sequences, consisting of a coiled-coil region that terminates in the eponymous, roughly 200-residue SUN domain, extends into the PNS. In mammals, there are five genes encoding orthodox SUN proteins ^27^. However, only two of these, *SUN1* and *SUN2*, are widely expressed.

SUN proteins typically function as transluminal tethers for ONM KASH (Klarsicht, ANC-1, SYNE, Homology) domain proteins ^4^. All members of the KASH family engage directly with available SUN domains through a short, roughly 30-residue, C-terminal sequence exposed to the PNS. The cytoplasmic N-terminal region of KASH family members binds components of the cytoskeleton. In this way, LINC complexes, spanning both nuclear membranes, are able to mechanically couple nuclear and cytoplasmic structures.

SUN proteins are clearly of ancient origin given their presence in all eukaryotic groups, where they have broadly similar roles. Indeed, glycosylated SUN homologues have even been recognised in archaeal cell walls. Based partly on such findings, Baum and Baum have suggested an intriguing “inside-out” model for the acquisition of nuclei and other membrane-bound compartments in eukaryotic cells ^28^. They propose that SUN proteins, and later LINC complexes, made a significant contribution to this key evolutionary process.

## LINC diversity

The role of LINC complexes in nuclear positioning and anchoring was first recognised in *Caenorhabditis elegans* by Starr and Han ^29, 30^. They showed that a large actin-binding protein, ANC-1, a prototypic KASH family member, was required for tethering hypodermal cell nuclei. Localisation of ANC-1 to the ONM was found to be dependent upon the SUN protein UNC-84. The single *C. elegans* lamin (lamin C) in turn was found to be essential for UNC-84 localisation ^31^. The inference was that UNC-84 together with ANC-1 formed a protein bridge that crossed the NE and that coupled the nuclear lamina to actin filaments. Similar SUN–KASH protein pairs had also been identified in both budding and fission yeast where they were shown to have roles in spindle pole assembly and organisation as well as in meiotic chromosome dynamics ^24, 25, 32, 33^. In *Drosophila*, LINC complexes consisting of the SUN protein Klaroid and the dynein-binding KASH protein Klarsicht were found to be essential for IKNM in the developing eye ^34, 35^. Other *Drosophila* LINC components contribute to muscle organisation ^36– 39^. Even in plants, nuclear positioning and movement are mediated by LINC complexes, featuring SUN family members that engage with functional homologues of fungal and animal KASH proteins ^40– 43^.

In mammals, there are six KASH proteins, of which five have well-characterised LINC complex functions ^4, 6, 44^. Two of these, Nesprin1 (Nesp1) and Nesprin2 (Nesp2), are encoded by a pair of complex genes featuring more than 100 exons ^45^. The largest isoforms of Nesp1 and 2, Nesprin1-Giant and Nesprin2-Giant (Nesp1G and Nesp2G), have masses of about 1 MDa and 800 kDa, respectively. Like ANC-1, each bears an N-terminal actin-binding site consisting of paired calponin-homology domains. In the case of at least Nesp2G, actin association is enhanced by an interaction with the actin nucleating protein formin, FHOD1, as well as with fascin, an actin bundling protein ^46, 47^. Both Nesp1 and 2 also contain binding sites for kinesin-1 and cytoplasmic dynein. In this way, Nesp1 and Nesp2, depending upon the isoform, can associate with the actin or microtubule systems or both. In developing muscle cells, Nesp1 is involved in the recruitment of centrosomal proteins to the NE, thereby co-opting the nucleus as a new microtubule-organising centre (MTOC) ^48, 49^. Similarly, a recent report ^50^ describes an association between Nesp1 and rootletin, a filament protein that forms ciliary rootlets. The implication is that Nesp1 may have a role in ciliary anchoring in a variety of cell types, including photoreceptors.

The third member of the family, Nesp3, binds plectin, a cytolinker molecule that provides a connection between the NE and the intermediate filament system ^51^. Nesp4 and KASH5 function as NE adaptors for kinesin-1 and cytoplasmic dynein, respectively ^52, 53^. Nesp4 is required for nuclear positioning in certain epithelial cells and is essential for viability of outer hair cells of the inner ear ^54^. Accordingly, Nesp4 mutations are linked to early-onset hearing loss ^54^. KASH5 is expressed primarily in meiotic cells and is responsible for telomere-led chromosome movements that culminate in homologous chromosome pairing ^53^. In *C. elegans*, ZYG-12, a dynein-binding KASH protein ^55^, has an analogous role ^56, 57^, while in budding and fission yeast, Kms1 and Csm4 perform roughly equivalent meiotic functions ^33, 58, 59^.

## LINC adjustments

Given the operational diversity of KASH proteins, it would seem logical that it is these that uniquely define LINC complex specificity. This view is reinforced by findings that mice deficient in either SUN1 or SUN2 are viable, indicating that these proteins, both widely (albeit not exclusively) co-expressed, are functionally redundant ^60^. However, recent articles have thrown a cat amongst this particular flock of pigeons. Gomes *et al*. have focused on the role of Nesp2G in nuclear repositioning during cell migration ^61^. It is well established that in migrating mouse 3T3 cells the MTOC becomes oriented towards the cell leading edge in front of the nucleus ^61^. However, the MTOC itself remains stationary at the cell centroid and instead it is the nucleus that actually moves rearward. This was demonstrated in classic scratch-wound assays on 3T3 monolayers. Although rearward movement occurs relative to the MTOC, the process itself is driven not by microtubules but rather by the retrograde flow of dorsal actin filaments that engage with LINC complexes in the NE. A consequence of this interaction is recruitment of Nesp2G, SUN2 and lamins into TAN (transmembrane actin-associated nuclear) lines subjacent to the actin filaments ^62, 63^. An additional ONM protein, Samp1 (NET5), also localised to TAN lines, is thought to contribute to the SUN2–lamin association ^64^.

To explore nuclear reorientation, Zhu *et al*. ^65^ devised a clever yet simple method. Employing the same scratch wound–type assay on cells grown on coverslips, nuclei were displaced by centrifugation. This was carried out by orienting the centrifugal field parallel to the coverslip surface and at right angles to the scratch in the cell monolayer. In this way, nuclei were moved to the front of cells on one side of the scratch while, on the other side, nuclei were displaced to the rear. The authors then followed the recovery of nuclei to their equilibrium positions. These experiments revealed that when the nucleus was displaced forwards it was returned to its correct location behind the MTOC in an actin- and myosin-dependent manner. This required LINC complexes containing Nesp2G and SUN2 and featured TAN line formation. However, when the nucleus was displaced rearwards, recovery was found to be dependent not on actin but on the microtubule system. In this case, nuclear movement was driven by cytoplasmic dynein. The identity of the dynein adaptor at the ONM was none other than Nesp2G. Evidently, cytoskeletal associations of Nesp2G may be modulated on the basis of the directionality of nuclear movement. However, the biggest surprise here was that for anterograde microtubule-dependent nuclear recovery, the LINC complex partner for Nesp2G was not SUN2 but rather SUN1. In the absence of SUN1, anterograde recovery was eliminated. The implication here is that the LINC complex partner of Nesp2G, either SUN1 or SUN2, is dictated by (or determines) the nature of its association with the cytoskeleton. The role of SUN proteins in either defining or reflecting the cytoskeletal engagement of Nesp2G is reinforced by the finding that the two SUN proteins can exert transdominant effects. Overexpression of SUN2 biases Nesp2G towards the actin system whereas overexpression of SUN1 has the reverse effect. The mechanisms underlying the differential effects of SUN1 versus SUN2 on the cytoskeletal interactions of Nesp2G are far from clear. However, they must in some way reflect the nature of the forces applied by actin versus microtubule systems and how they are accommodated by the two SUN proteins. Recent work from Cain *et al*. ^66^ also points to mechanistic differences in the way that LINC complexes may transmit sustained forces generated by the actin cytoskeleton versus transient forces generated by the microtubule system.

## Something new under the SUN

The original structural analyses of SUN–KASH complexes revealed that SUN2 SUN domains are organised as homotrimers ^67, 68^. *In vivo* this would involve the association of three SUN2 monomers by the formation of an extended triple-helical coiled coil that links the SUN2 transmembrane sequences to the terminal SUN domains. Association with a Nesp1 or Nesp2 KASH domain occurs mainly at contact sites between SUN domains within each trimer. This interface is formed where an anti-parallel beta-structure (the “KASH-lid”) extending from one SUN domain overlaps with the core structure of its neighbor. LINC complex assembly involves 18 to 23 amino acids at the KASH domain C-terminus. These are organised in an extended conformation and are accommodated largely within a groove beneath the KASH-lid. However, the four terminal amino acids of the KASH domain, comprising a PPPX (P is proline and X is any amino acid) motif, fit into a discrete pocket within a single SUN monomer. Integrity of this motif is crucial for the SUN–KASH interaction. Extension of this sequence by only a single alanine abolishes association. The implication is that the SUN–KASH interaction is initiated by the PPPX tetrapeptide followed by “zippering-up” of the KASH sequence within the binding groove. Additional KASH residues are also important: a tyrosine at position -7 (Y(-)7) and a cysteine at position -23. The latter is able to form a disulphide bond with a conserved cysteine residue (C563 in human SUN2) within the SUN domain. Cain *et al*. examined the role of these different KASH residues in *C. elegans* LINC complexes *in vivo* ^66^. In particular, they explored interactions of the SUN protein UNC-84 with its two partners: ANC-1 and UNC-83. The latter is a KASH protein that binds kinesin-1 and cytoplasmic dynein and is required for nuclear migration in hypodermal precursor cells ^69– 71^. As we have seen, ANC-1 is required for actin-dependent nuclear anchoring in the hypodermis ^29^. For both ANC-1 and UNC-83, integrity of the C-terminus is paramount for productive association with UNC-84. The Y(-)7 in UNC-83 also proves to be essential. While still localising to the NE, an alanine substitution at this position renders UNC-83 incapable of supporting nuclear migration. The inference is that interaction with UNC-84 is sufficiently weakened so that it cannot withstand application of forces encountered during kinesin-driven nuclear movement. In contrast, disulphide bond formation is not required. Indeed, UNC-83 has a foreshortened KASH domain that lacks the key cysteine residue. The reverse is found to be true for ANC-1. Y(-)7 is dispensable, while the cysteine at -23 is absolutely required for nuclear anchoring. The suggestion is that for short-term processes such as UNC-83 and microtubule-driven migration, maintenance of SUN–KASH interactions by a disulphide bond may be unnecessary. By contrast, long-term anchoring via actin may require disulphide-dependent LINC complex stabilisation. This notion makes some sense since the mammalian dynein-binding KASH protein KASH5, which has only a transient function in meiotic cells, like UNC-83 has a foreshortened KASH domain and lacks the cysteine at -23. The role of disulphide bond formation in the stabilisation of SUN2–Nesp2 association is brought further into focus in molecular dynamics studies ^66^. Loss of this bond results in significantly weakened interactions, and application of pulling forces to the Nesp2 KASH peptide causes its partial detachment. Moreover, both the KASH peptide and the SUN2 KASH-lid become stretched out to accommodate the applied forces. With the disulphide bond intact, however, forces are transmitted beyond the SUN–KASH interface and instead are relieved by stretching of the triple-helical coiled coil.

It is obvious that diverse KASH proteins display subtly different associations with their cognate SUN proteins. However, this cannot easily explain observations that the cytoskeletal interactions of Nesp2 are reflected in its discrimination between SUN1 and SUN2 ^65^. It seems likely, however, that the key to this puzzle lies in how forces across LINC complexes are dissipated. Studies by Nie *et al*. and Xu *et al.* suggest that the triple-helical coiled coil may play a significant role in the regulation of SUN–KASH interactions ^72, 73^. Structural analyses reveal that the lumenal coiled-coil domain is actually formed from two separate helical segments, CC1 and CC2, which are connected by a flexible linker. By the same token, CC2, which lies closest to the SUN domain, is itself formed from three shorter helices. These helices, in addition to facilitating trimerisation of SUN2 monomers, can adopt an alternative structure in which they form an intra-chain triple-helical bundle. In this conformation, CC2 is able to associate with the SUN domain KASH-lid, thereby effectively blocking LINC complex formation. In this way, CC2 functions as an auto-inhibitory domain (AID). Clearly, CC2 must be able to switch reversibly between its inhibitory conformation and its extended conformation permissive for trimerisation and KASH binding. What is not clear is whether this switching may still occur after trimer formation ^74^. If it can occur, then it opens up a range of regulatory possibilities. In one such model, tension applied to CC2 via a bound KASH domain should favour its extended permissive conformation over its inhibitory conformation. Given that there is some divergence between SUN1 and SUN2 CC2/AID sequences, it is possible that one or the other may be more prone to switching to the AID conformation. Certainly, Hennen *et al*. ^75^ and Jahed *et al*. ^76^ have documented differences in the limitations of SUN1 versus SUN2 homo-oligomerisation. As an example, the actin system may be less effective at retaining SUN1 in its permissive conformation when compared with the microtubule system. Thus, Nesp2G, when bound to actin, may be biased away from SUN1. Conversely, Nesp2G, when engaged with kinesin-1, may be biased towards SUN1 because of its ability to prevent auto-inhibition by CC2. This model would be consistent with observations that in mammalian systems, all microtubule-associated LINC complexes seem to be based around SUN1, regardless of whether they contain Nesp2, Nesp4 or KASH5. It is also certain that additional factors, such as the AAA ^+^ (ATPase associated with various cellular activities) protein TorsinA, contribute to these various SUN–KASH associations ^77^. Intriguingly, a recent report described a *Drosophila* ONM protein, Kuduk (Kud), which associates either directly or indirectly with KASH proteins ^78^. Kud appears to suppress NE anchorage of KASH proteins lacking cytoskeletal interactions. In this way, Kud and its mammalian homologue TMEM258 may function as a quality control or chaperone for the assembly of LINC complexes that are engaged with the cytoskeleton.

The scheme outlined here, which involves SUN auto-inhibition and which draws on work from several laboratories, cannot be the whole story. The model, as it stands, cannot easily explain reciprocal transdominant effects of SUN1 versus SUN2 in Nesp2G-dependent nuclear positioning ^65^. However, taken together, all of these recent biophysical and cell biological studies have hinted at previously unappreciated levels of LINC complex adaptation. There is no question that these may impact diverse aspects of cell function from mechanotransduction to migration and will open new avenues of research activities.

## References

[1] Bone CR Starr DA: Nuclear migration events throughout development. *J Cell Sci.* 2016;129(10):1951–61. 10.1242/jcs.179788 27182060 PMC5998656

[2] Maniotis AJ Chen CS Ingber DE: Demonstration of mechanical connections between integrins, cytoskeletal filaments, and nucleoplasm that stabilize nuclear structure. *Proc Natl Acad Sci U S A.* 1997;94(3):849–54. 10.1073/pnas.94.3.849 9023345 PMC19602

[3] Lombardi ML Jaalouk DE Shanahan CM: The interaction between nesprins and sun proteins at the nuclear envelope is critical for force transmission between the nucleus and cytoskeleton. *J Biol Chem.* 2011;286(30):26743–53. 10.1074/jbc.M111.233700 21652697 PMC3143636

[4] Chang W Worman HJ Gundersen GG: Accessorizing and anchoring the LINC complex for multifunctionality. *J Cell Biol.* 2015;208(1):11–22. 10.1083/jcb.201409047 25559183 PMC4284225

[5] Gundersen GG Worman HJ: Nuclear positioning. *Cell.* 2013;152(6):1376–89. 10.1016/j.cell.2013.02.031 23498944 PMC3626264

[6] Lee YL Burke B: LINC complexes and nuclear positioning. *Semin Cell Dev Biol.* 2018;82:67–76. 10.1016/j.semcdb.2017.11.008 29191370

[7] Crisp M Burke B: The nuclear envelope as an integrator of nuclear and cytoplasmic architecture. *FEBS Lett.* 2008;582(14):2023–32. 10.1016/j.febslet.2008.05.001 18474238

[8] Wilson KL Dawson SC: Evolution: functional evolution of nuclear structure. *J Cell Biol.* 2011;195(2):171–81. 10.1083/jcb.201103171 22006947 PMC3198171

[9] Burke B Stewart CL: The nuclear lamins: flexibility in function. *Nat Rev Mol Cell Biol.* 2013;14(1):13–24. 10.1038/nrm3488 23212477

[10] Gerace L Huber MD: Nuclear lamina at the crossroads of the cytoplasm and nucleus. *J Struct Biol.* 2012;177(1):24–31. 10.1016/j.jsb.2011.11.007 22126840 PMC3261324

[11] Busson S Dujardin D Moreau A: Dynein and dynactin are localized to astral microtubules and at cortical sites in mitotic epithelial cells. *Curr Biol.* 1998;8(9):541–4. 10.1016/S0960-9822(98)70208-8 9560347

[12] Gönczy P Pichler S Kirkham M: Cytoplasmic dynein is required for distinct aspects of MTOC positioning, including centrosome separation, in the one cell stage *Caenorhabditis elegans* embryo. *J Cell Biol.* 1999;147(1):135–50. 10.1083/jcb.147.1.135 10508861 PMC2164971

[13] Reinsch S Karsenti E: Movement of nuclei along microtubules in *Xenopus* egg extracts. *Curr Biol.* 1997;7(3):211–4. 10.1016/S0960-9822(97)70092-7 9395409

[14] Salina D Bodoor K Eckley DM: Cytoplasmic dynein as a facilitator of nuclear envelope breakdown. *Cell.* 2002;108(1):97–107. 10.1016/S0092-8674(01)00628-6 11792324

[15] Beaudouin J Gerlich D Daigle N: Nuclear envelope breakdown proceeds by microtubule-induced tearing of the lamina. *Cell.* 2002;108(1):83–96. 10.1016/S0092-8674(01)00627-4 11792323

[16] Hu DJ Baffet AD Nayak T: Dynein recruitment to nuclear pores activates apical nuclear migration and mitotic entry in brain progenitor cells. *Cell.* 2013;154(6):1300–13. 10.1016/j.cell.2013.08.024 24034252 PMC3822917

[17] Splinter D Tanenbaum ME Lindqvist A: Bicaudal D2, dynein, and kinesin-1 associate with nuclear pore complexes and regulate centrosome and nuclear positioning during mitotic entry. *PLoS Biol.* 2010;8(4):e1000350. 10.1371/journal.pbio.1000350 20386726 PMC2850381

[18] Meier I: LINCing the eukaryotic tree of life - towards a broad evolutionary comparison of nucleocytoplasmic bridging complexes. *J Cell Sci.* 2016;129(19):3523–31. 10.1242/jcs.186700 27591260

[19] Zhou X Meier I: How plants LINC the SUN to KASH. *Nucleus.* 2013;4(3):206–15. 10.4161/nucl.24088 23680964 PMC3720751

[20] Crisp M Liu Q Roux K: Coupling of the nucleus and cytoplasm: role of the LINC complex. *J Cell Biol.* 2006;172(1):41–53. 10.1083/jcb.200509124 16380439 PMC2063530

[21] Haque F Lloyd DJ Smallwood DT: SUN1 interacts with nuclear lamin A and cytoplasmic nesprins to provide a physical connection between the nuclear lamina and the cytoskeleton. *Mol Cell Biol.* 2006;26(10):3738–51. 10.1128/MCB.26.10.3738-3751.2006 16648470 PMC1488999

[22] Hasan S Güttinger S Mühlhäusser P: Nuclear envelope localization of human UNC84A does not require nuclear lamins. *FEBS Lett.* 2006;580(5):1263–8. 10.1016/j.febslet.2006.01.039 16445915

[23] Padmakumar VC Libotte T Lu W: The inner nuclear membrane protein Sun1 mediates the anchorage of Nesprin-2 to the nuclear envelope. *J Cell Sci.* 2005;118(Pt 15):3419–30. 10.1242/jcs.02471 16079285

[24] Jaspersen SL Giddings TH Jr Winey M: Mps3p is a novel component of the yeast spindle pole body that interacts with the yeast centrin homologue Cdc31p. *J Cell Biol.* 2002;159(6):945–56. 10.1083/jcb.200208169 12486115 PMC2173979

[25] Jaspersen SL Martin AE Glazko G: The Sad1-UNC-84 homology domain in Mps3 interacts with Mps2 to connect the spindle pole body with the nuclear envelope. *J Cell Biol.* 2006;174(5):665–75. 10.1083/jcb.200601062 16923827 PMC2064310

[26] King MC Drivas TG Blobel G: A network of nuclear envelope membrane proteins linking centromeres to microtubules. *Cell.* 2008;134(3):427–38. 10.1016/j.cell.2008.06.022 18692466 PMC2617791

[27] Liu Q Pante N Misteli T: Functional association of Sun1 with nuclear pore complexes. *J Cell Biol.* 2007;178(5):785–98. 10.1083/jcb.200704108 17724119 PMC2064544

[28] Baum DA Baum B: An inside-out origin for the eukaryotic cell. *BMC Biol.* 2014;12:76. 10.1186/s12915-014-0076-2 25350791 PMC4210606

[29] Starr DA Han M: Role of ANC-1 in tethering nuclei to the actin cytoskeleton. *Science.* 2002;298(5592):406–9. 10.1126/science.1075119 12169658

[30] Starr DA Han M: ANChors away: an actin based mechanism of nuclear positioning. *J Cell Sci.* 2003;116(Pt 2):211–6. 10.1242/jcs.00248 12482907

[31] Lee KK Starr D Cohen M: Lamin-dependent localization of UNC-84, a protein required for nuclear migration in *Caenorhabditis elegans*. *Mol Biol Cell.* 2002;13(3):892–901. 10.1091/mbc.01-06-0294 11907270 PMC99607

[32] Hiraoka Y Dernburg AF: The SUN rises on meiotic chromosome dynamics. *Dev Cell.* 2009;17(5):598–605. 10.1016/j.devcel.2009.10.014 19922865

[33] Shimanuki M Miki F Ding DQ: A novel fission yeast gene, *kms1* ^+^, is required for the formation of meiotic prophase-specific nuclear architecture. *Mol Gen Genet.* 1997;254(3):238–49. 10.1007/s004380050412 9150257

[34] Mosley-Bishop KL Li Q Patterson L: Molecular analysis of the *klarsicht* gene and its role in nuclear migration within differentiating cells of the *Drosophila* eye. *Curr Biol.* 1999;9(21):1211–20. 10.1016/S0960-9822(99)80501-6 10556085

[35] Kracklauer MP Banks SM Xie X: Drosophila *klaroid* encodes a SUN domain protein required for Klarsicht localization to the nuclear envelope and nuclear migration in the eye. *Fly (Austin).* 2014;1(2):75–85. 10.4161/fly.4254 18820457

[36] Volk T: A new member of the spectrin superfamily may participate in the formation of embryonic muscle attachments in *Drosophila*. *Development.* 1992;116(3):721–30. 1289062 10.1242/dev.116.3.721

[37] Volk T: Positioning nuclei within the cytoplasm of striated muscle fiber: cooperation between microtubules and KASH proteins. *Nucleus.* 2013;4(1):18–22. 10.4161/nucl.23086 23211643 PMC3585022

[38] Wang S Reuveny A Volk T: Nesprin provides elastic properties to muscle nuclei by cooperating with spectraplakin and EB1. *J Cell Biol.* 2015;209(4):529–38. 10.1083/jcb.201408098 26008743 PMC4442817

[39] Elhanany-Tamir H Yu YV Shnayder M: Organelle positioning in muscles requires cooperation between two KASH proteins and microtubules. *J Cell Biol.* 2012;198(5):833–46. 10.1083/jcb.201204102 22927463 PMC3432764

[40] Graumann K Runions J Evans DE: Characterization of SUN-domain proteins at the higher plant nuclear envelope. *Plant J.* 2010;61(1):134–44. 10.1111/j.1365-313X.2009.04038.x 19807882

[41] Graumann K Vanrobays E Tutois S: Characterization of two distinct subfamilies of SUN-domain proteins in *Arabidopsis* and their interactions with the novel KASH-domain protein AtTIK. *J Exp Bot.* 2014;65(22):6499–512. 10.1093/jxb/eru368 25217773

[42] Zhou X Graumann K Evans DE: Novel plant SUN-KASH bridges are involved in RanGAP anchoring and nuclear shape determination. *J Cell Biol.* 2012;196(2):203–11. 10.1083/jcb.201108098 22270916 PMC3265956

[43] Zhou X Graumann K Wirthmueller L: Identification of unique SUN-interacting nuclear envelope proteins with diverse functions in plants. *J Cell Biol.* 2014;205(5):677–92. 10.1083/jcb.201401138 24891605 PMC4050730

[44] Horn HF: LINC complex proteins in development and disease. *Curr Top Dev Biol.* 2014;109:287–321. 10.1016/B978-0-12-397920-9.00004-4 24947240

[45] Zhang Q Skepper JN Yang F: Nesprins: a novel family of spectrin-repeat-containing proteins that localize to the nuclear membrane in multiple tissues. *J Cell Sci.* 2001;114(Pt 24):4485–98. 11792814 10.1242/jcs.114.24.4485

[46] Kutscheidt S Zhu R Antoku S: FHOD1 interaction with nesprin-2G mediates TAN line formation and nuclear movement. *Nat Cell Biol.* 2014;16(7):708–15. 10.1038/ncb2981 24880667 PMC4113092

[47] Jayo A Malboubi M Antoku S: Fascin Regulates Nuclear Movement and Deformation in Migrating Cells. *Dev Cell.* 2016;38(4):371–83. 10.1016/j.devcel.2016.07.021 27554857 PMC4997957

[48] Espigat-Georger A Dyachuk V Chemin C: Nuclear alignment in myotubes requires centrosome proteins recruited by nesprin-1. *J Cell Sci.* 2016;129(22):4227–37. 10.1242/jcs.191767 27802164

[49] Gimpel P Lee YL Sobota RM: Nesprin-1α-Dependent Microtubule Nucleation from the Nuclear Envelope via Akap450 Is Necessary for Nuclear Positioning in Muscle Cells. *Curr Biol.* 2017;27(19):2999–3009.e9. 10.1016/j.cub.2017.08.031 28966089 PMC5640514

[50] Potter C Zhu W Razafsky D: Multiple Isoforms of Nesprin1 Are Integral Components of Ciliary Rootlets. *Curr Biol.* 2017;27(13):2014–2022.e6. 10.1016/j.cub.2017.05.066 28625779 PMC5546243

[51] Ketema M Kreft M Secades P: Nesprin-3 connects plectin and vimentin to the nuclear envelope of Sertoli cells but is not required for Sertoli cell function in spermatogenesis. *Mol Biol Cell.* 2013;24(15):2454–66. 10.1091/mbc.E13-02-0100 23761073 PMC3727937

[52] Roux KJ Crisp ML Liu Q: Nesprin 4 is an outer nuclear membrane protein that can induce kinesin-mediated cell polarization. *Proc Natl Acad Sci U S A.* 2009;106(7):2194–9. 10.1073/pnas.0808602106 19164528 PMC2650131

[53] Horn HF Kim DI Wright GD: A mammalian KASH domain protein coupling meiotic chromosomes to the cytoskeleton. *J Cell Biol.* 2013;202(7):1023–39. 10.1083/jcb.201304004 24062341 PMC3787381

[54] Horn HF Brownstein Z Lenz DR: The LINC complex is essential for hearing. *J Clin Invest.* 2013;123(2):740–50. 10.1172/JCI66911 23348741 PMC3561815

[55] Malone CJ Misner L Le Bot N: The *C. elegans* hook protein, ZYG-12, mediates the essential attachment between the centrosome and nucleus. *Cell.* 2003;115(7):825–36. 10.1016/S0092-8674(03)00985-1 14697201

[56] Sato A Isaac B Phillips CM: Cytoskeletal forces span the nuclear envelope to coordinate meiotic chromosome pairing and synapsis. *Cell.* 2009;139(5):907–19. 10.1016/j.cell.2009.10.039 19913287 PMC2825574

[57] Penkner AM Fridkin A Gloggnitzer J: Meiotic chromosome homology search involves modifications of the nuclear envelope protein Matefin/SUN-1. *Cell.* 2009;139(5):920–33. 10.1016/j.cell.2009.10.045 19913286

[58] Kosaka H Shinohara M Shinohara A: Csm4-dependent telomere movement on nuclear envelope promotes meiotic recombination. *PLoS Genet.* 2008;4(9):e1000196. 10.1371/journal.pgen.1000196 18818742 PMC2533704

[59] Conrad MN Lee C-Y Chao G: Rapid telomere movement in meiotic prophase is promoted by *NDJ1*, *MPS3*, and *CSM4* and is modulated by recombination. *Cell.* 2008;133(7):1175–87. 10.1016/j.cell.2008.04.047 18585352

[60] Lei K Zhang X Ding X: SUN1 and SUN2 play critical but partially redundant roles in anchoring nuclei in skeletal muscle cells in mice. *Proc Natl Acad Sci U S A.* 2009;106(25):10207–12. 10.1073/pnas.0812037106 19509342 PMC2700906

[61] Gomes ER Jani S Gundersen GG: Nuclear movement regulated by Cdc42, MRCK, myosin, and actin flow establishes MTOC polarization in migrating cells. *Cell.* 2005;121(3):451–63. 10.1016/j.cell.2005.02.022 15882626

[62] Luxton GW Gomes ER Folker ES: Linear arrays of nuclear envelope proteins harness retrograde actin flow for nuclear movement. *Science.* 2010;329(5994):956–9. 10.1126/science.1189072 20724637 PMC3938394

[63] Luxton GW Gomes ER Folker ES: TAN lines: a novel nuclear envelope structure involved in nuclear positioning. *Nucleus.* 2011;2(3):173–81. 10.4161/nucl.2.3.16243 21818410 PMC3149877

[64] Borrego-Pinto J Jegou T Osorio DS: Samp1 is a component of TAN lines and is required for nuclear movement. *J Cell Sci.* 2012;125(Pt 5):1099–105. 10.1242/jcs.087049 22349700

[65] Zhu R Antoku S Gundersen GG: Centrifugal Displacement of Nuclei Reveals Multiple LINC Complex Mechanisms for Homeostatic Nuclear Positioning. *Curr Biol.* 2017;27(20):3097–3110.e5. 10.1016/j.cub.2017.08.073 28988861 PMC5688853

[66] Cain NE Jahed Z Schoenhofen A: Conserved SUN-KASH Interfaces Mediate LINC Complex-Dependent Nuclear Movement and Positioning. *Curr Biol.* 2018;28(19):3086–3097.e4. 10.1016/j.cub.2018.08.001 30245107 PMC6219386

[67] Sosa BA Kutay U Schwartz TU: Structural insights into LINC complexes. *Curr Opin Struct Biol.* 2013;23(2):285–91. 10.1016/j.sbi.2013.03.005 23597672 PMC4077334

[68] Zhou Z Du X Cai Z: Structure of Sad1-UNC84 homology (SUN) domain defines features of molecular bridge in nuclear envelope. *J Biol Chem.* 2012;287(8):5317–26. 10.1074/jbc.M111.304543 22170055 PMC3285312

[69] Fridolfsson HN Ly N Meyerzon M: UNC-83 coordinates kinesin-1 and dynein activities at the nuclear envelope during nuclear migration. *Dev Biol.* 2010;338(2):237–50. 10.1016/j.ydbio.2009.12.004 20005871 PMC2826220

[70] Meyerzon M Fridolfsson HN Ly N: UNC-83 is a nuclear-specific cargo adaptor for kinesin-1-mediated nuclear migration. *Development.* 2009;136(16):2725–33. 10.1242/dev.038596 19605495 PMC2730402

[71] Starr DA Hermann GJ Malone CJ: unc-83 encodes a novel component of the nuclear envelope and is essential for proper nuclear migration. *Development.* 2001;128(24):5039–50. 11748140 10.1242/dev.128.24.5039

[72] Nie S Ke H Gao F: Coiled-Coil Domains of SUN Proteins as Intrinsic Dynamic Regulators. *Structure.* 2016;24(1):80–91. 10.1016/j.str.2015.10.024 26688217

[73] Xu Y Li W Ke H: Structural conservation of the autoinhibitory domain in SUN proteins. *Biochem Biophys Res Commun.* 2018;496(4):1337–43. 10.1016/j.bbrc.2018.02.015 29408528

[74] Jahed Z Vu UT Fadavi D: A molecular model for LINC complex regulation: activation of SUN2 for KASH binding. *Mol Biol Cell.* 2018;29(16):2012–23. 10.1091/mbc.E18-04-0266 29995584 PMC6232973

[75] Hennen J Saunders CA Mueller JD: Fluorescence fluctuation spectroscopy reveals differential SUN protein oligomerization in living cells. *Mol Biol Cell.* 2018;29(9):1003–11. 10.1091/mbc.E17-04-0233 29514929 PMC5921568

[76] Jahed Z Fadavi D Vu UT: Molecular Insights into the Mechanisms of SUN1 Oligomerization in the Nuclear Envelope. *Biophys J.* 2018;114(5):1190–203. 10.1016/j.bpj.2018.01.015 29539404 PMC5883562

[77] Saunders CA Harris NJ Willey PT: TorsinA controls TAN line assembly and the retrograde flow of dorsal perinuclear actin cables during rearward nuclear movement. *J Cell Biol.* 2017;216(3):657–74. 10.1083/jcb.201507113 28242745 PMC5350507

[78] Ding ZY Wang YH Huang YC: Outer nuclear membrane protein Kuduk modulates the LINC complex and nuclear envelope architecture. *J Cell Biol.* 2017;216(9):2827–2841. 10.1083/jcb.201606043 28716842 PMC5584142

